# Prophylactic Avoidance of Hazardous Prey by the Ant Host *Myrmica rubra*

**DOI:** 10.3390/insects11070444

**Published:** 2020-07-14

**Authors:** Hugo Pereira, Claire Detrain

**Affiliations:** Unit of Social Ecology, CP 231, Université Libre de Bruxelles, Boulevard du Triomphe, 1050 Brussels, Belgium; cdetrain@ulb.ac.be

**Keywords:** avoidance-foraging, grooming, *Metarhizium brunneum*, *Myrmica rubra*, social immunity

## Abstract

Ants are the hosts of many microorganisms, including pathogens that are incidentally brought inside the nest by foragers. This is particularly true for scavenging species, which collect hazardous food such as dead insects. Foragers limit sanitary risks by not retrieving highly infectious prey releasing entomopathogenic fungal spores. This study investigates whether similar prophylactic strategies are also developed for food associated with weak or delayed risks of fungal contamination. We compared, in *Myrmica rubra* ant colonies, the retrieval dynamics of dead flies that were (1) conidia-free, (2) covered with a low amount of *Metarhizium brunneum* entomopathogenic conidia or (3) recently fungus-killed but not yet sporulating. Foragers mostly avoided fungus-killed prey and delayed the retrieval of conidia-covered flies. A second sanitary filter occurred inside the nest through a careful inspection of the retrieved prey. Ultimately, ants mostly consumed conidia-free and conidia-covered flies, but they relocated and discarded all fungus-killed prey outside of the nest. Our study confirms that, as a host of generalist entomopathogenic fungi, *Myrmica rubra* ants have developed a prophylactic avoidance and a differential management of prey depending on their infectious potential. We discuss the functional value as well as the possible cues underlying pathogen avoidance and prey discrimination in ants.

## 1. Introduction

The ecological success of eusocial insects such as ants stems from a high level of cooperation between nestmates. While a few individuals ensure the reproduction, workers are collectively engaged in carrying out different daily tasks such as foraging, nest defence, waste management, nest maintenance and brood care [1]. The efficiency of this work organization contributes to the survival of the whole colony and its development [2]. Cooperation in task performance requires that workers frequently come into contact with each other inside the confined environment of their nest. Coupled with a high level of genetic relatedness between nestmates, such a high rate of interactions increases the risk of disease outbreak resulting from a contamination by hazardous entomopathogens. In the absence of any strategy to counter sanitary threats, the proliferation of a generalist or species-specific pathogen will reduce the activity level of colony members, increase their mortality rate and eventually lead to the collapse of the whole nest population [3,4].

For this reason, besides the individual immunity which protects insects against macro- and micro-parasites [5,6], eusocial insects have developed a variety of collective sanitary strategies, called “social immunity”. Social immunity aims to reduce the uptake of pathogens from the external environment, to limit their development inside the nest and to increase resistance to infectious sources among colony members [7,8,9,10]. Social immunity is characterized by adaptive cooperative social defences that evolved at the physiological, behavioural and organisational levels in order to limit sanitary threats on colony survival (ants: [11], termites: [12,13], bees: [14], wasps: [15], reviewed in [10,16,17]). A major challenge for insect societies is to lower the risk of horizontal transmission of pathogens between nestmates. This is namely achieved through an organisational immunity which basically relies on a spatial and social segregation between potential vectors of pathogens such as foragers and individuals of the utmost importance for the colony future, such as the queen or the larvae (ants: [18], bees: [19], reviewed in [20]). Such a hygienic distancing also applies for organic waste (e.g., food remains, faeces) [21,22] or dead nestmates (ants: [23,24,25], bees: [26,27], bumble bees: [28], reviewed in: [29,30]) which are actively discarded by nest cleaners and undertakers. Likewise, moribund workers, close to death, self-exclude from their colony by exiting the nest and die in social isolation far from their nestmates (ants: [31,32], bees: [33]).

The abovementioned mechanisms of organizational immunity demonstrate that the nest can be seen as a homeostatic location where there is a permanent and more intensive sanitary control than in the external environment [34]. To maintain a satisfactory level of nest hygiene, a first line of prophylaxis consists of limiting the entry of potentially hazardous items into the nest. One of the main entry pathways for pathogens is through the back-and-forth journeys of foragers which may incidentally bring in infectious agents on their bodies or on the retrieved food items. Thus, foragers represent a critical sanitary filter by choosing not to retrieve hazardous food items inside the nest. Previous studies have shown that ant foragers are actually able to discriminate a potentially infectious food from a non-contaminated one. For example, some ant or termite species do not cannibalise fungus-killed nestmates (ants: [35], termites: [36]). Likewise, several ant species avoid being exposed to entomopathogenic fungi by not collecting items covered with a high amount of *Metarhizium anisopliae* fungus conidia [37] or sporulating cadavers [38]. Such a discrimination may result from the perception of volatile chemical compounds released by the pathogen itself [39] or by its interaction with the insect host [40].

Interestingly, there are also a few counter-examples that report a lack of discrimination of infected food items [41] or even an attraction towards a fungus-contaminated environment [42,43]. Within the same ant species, colonies may also differ in their response to pathogens, with some avoiding locations contaminated by fungal spores and others preferentially digging their nest inside a spore-containing soil [44]. In a recent study on the red ant *Myrmica rubra* [38], we found that the retrieval of prey differed depending on its state of infection. While *M. rubra* foragers retrieved most of the prey that were simply covered with *M. bruneum* fungal conidia, they were less likely to bring back an insect that recently died from fungus infection and did not transport any sporulating prey at all. These findings suggest that the strategy of pathogen avoidance used by ants is more complex than a simple rule-of thumb. 

*Myrmica rubra* is an opportunistic omnivorous species whose workers collect aphid honeydew but also regularly forage on living or dead insects [45]. Since cadavers or dying insects can be infected by pathogens, these food items may represent a sanitary risk for the ant consumers [4]. As a potential host for generalist entomopathogens, ant species with a scavenger activity are assumed to have developed a set of physiological or behavioural defences against food-related contamination. In this context, foragers represent a first line of food quality auditors. For example, by simply avoiding retrieving sporulating prey, *Myrmica rubra* foragers prevent the uptake of a high load of pathogens inside the nest [38]. However, we still do not know to what extent this first line of protection is permeable to food items that are weakly infectious or with a delayed pathogenicity—i.e., infected but not yet infectious items. When such items are retrieved by foragers inside the nest, one may wonder whether additional sanitary filters inside the nest will ensure the control of food safety before feeding larvae. This is a key issue, since the soft and weakly sclerotized cuticle of larvae make them highly susceptible to contact-transmitted parasites, such as the opportunistic entomopathogenic fungus, *Metarhizium brunneum* [46]. For example, gatekeepers or nurses could deny access to contaminated food items or could reject them outside of the nest. As an alternative to active discarding, workers could simply lose interest in contaminated food items, handle them as wastes and/or leave them in remote nest chambers. 

In the present study, we investigate whether *M. rubra* colonies can detect sanitary risks early by avoiding retrieving and/or consuming prey with a weak or delayed infectiousness. We will compare the retrieval dynamics as well as the within-nest handling of dead flies that were (1) conidia-free, (2) covered with a low amount of *Metarhizium brunneum* conidia or (3) killed by the fungus but not yet sporulating. We will look at the sanitary filters at work in ant colonies while foraging on prey. Finally, we will see whether ant colonies alter their foraging behaviour depending on whether they previously experienced the retrieval of fungus-contaminated prey. 

## 2. Material and Methods

### 2.1. Studied Ant Species and Rearing of Ant Colonies

*Myrmica rubra* is a polydomous, polygynous and monomorphic ant species that is common in European temperate areas. This ant species lives in semi-humid conditions and can be found in biotopes such as semi-open grasslands. *Myrmica rubra* nests are dug in various substrates, such as in ground under stones, inside rotting wood, and among the roots of nettles and bramble bushes [47]. 

We collected seven queenright and broodright colonies of *Myrmica rubra,* in Belgium, from the localities of Sambreville (50°25′59.62″ N; 4°37′22.12″ E), Falisolle (50°25′11.99″ N; 4°37′50.41″ E) and Brussels (ULB university campus; 50°49′05.1″ N; 4°24′01.6″ E) during the summer of 2018 and 2019. Colonies were kept in the laboratory in a thermo-regulated room at 21 ± 2 °C, around 50% relative humidity and a constant light-dark 12 h period. Each colony was placed in a box (22 × 14 × 9 cm) that was used by ants as a foraging area and whose walls were coated with polytetrafluoroethylene (Fluon; Whitford, UK) to prevent ants from escaping. Ant colonies settled in test tubes that were half-filled with a water reservoir plugged by a humid cotton wool and that were covered with a red filter to provide darkness to the ants. Colonies were provided with water, sucrose solution (0.3 M) ad libitum as well as mealworms (*Tenebrio molitor*) twice per week as a source of proteins and lipids.

### 2.2. Rearing of Fruit Flies

The prey used in the experiments were *Drosophila melanogaster* fruit flies that belonged to the “vestigial wings” phenotype. These fruit flies were reared on a home-made food substrate (79% applesauce, 3% oat bran, 12% mashed potatoes in snowflakes, 5% white vinegar) at a room temperature of 21 °C and a 50% relative humidity. We dropped raffia strings on the mixture in order to facilitate the pupation of larvae. We renewed the food substrate once per month.

### 2.3. Metarhizium Brunneum Entomopathogenic Fungus

To contaminate the prey fly, we used the entomopathogenic fungus *Metarhizium brunneum* (strain F52) from Novozymes Bayer^TM^. This fungus can kill over 200 different insect species, including flies such as *Drosophila melanogaster* fruit fly [38] or ants such the red ant *M. rubra* species [48]. Fungal conidia of *M. brunneum* occur in the natural habitat of *M. rubra* ant colonies [49] and have been used both as a control agent of pest ants in the field [50] and as reference pathogens in basic research on social immunity (ants: [25,38,48,51], termites: [52,53], bees: [54]). As regards the developmental cycle of the fungus, once conidia come in contact with the insect and attach themselves on its body surface, appressiora begin to grow and to pierce the insect cuticle. The fungal mycelium then spreads inside the host haemocoel and eventually causes the death of the host insect. Several days after the insect’s death, the fungus starts sporulating outside the corpse and releasing new conidia into the environment [55].

### 2.4. Tested Conditions

We tested ant responses to three types of prey, differing by their level of infectiousness. In the first condition, we offered to ant colonies 10 flies that had been killed by exposure to cold (−10 °C) around one hour before the experiment and that were conidia-free (Control condition: Ctrl).

In the second condition, the 10 offered flies were prey first killed by exposure to cold and then were covered with a low amount of around 5000 conidia per fly. Conidia were applied by delivering 1 µL of a suspension of conidia (Low amount of Conidia: LC) one hour before the experiment in order to let the solution dry on the insect cuticle. To obtain a suspension of conidia of known concentration, we put a sporulating corpse in 500 µL of Triton X-20 solution (0.05%) that was centrifugated in a Eppendorf during 5 min at 6000 rounds per minute. We cautiously removed the supernatant and we added 200 µL of Tween 20 (0.05%). We scattered the pellet and homogenised the solution by shaking the Eppendorf during three phases of 10 s with a vortex mixer. We used a microscope with a Thoma’s cell to estimate the concentration of this initial suspension of conidia and we diluted it with Tween 20 in order to reach the desired concentration of conidia.

For the last condition, we offered to ant colonies 10 flies that had been killed by the fungus less than 24 h ago and were not yet sporulating (Fungus-Killed: Fkill). To do so, we infected flies with the fungus by vortexing them together with a sporulating corpse, four times for five seconds at a speed of 1500 rounds/min, as described in the protocol of Leclerc and Detrain [48]. This process ensures that a large amount of conidia will be attached to the insect cuticle and therefore will induce a high mortality rate (87%) among the contaminated fruit flies. The infected flies usually died at day-5 post-infection and were used for the experiment within 24 h following their death. Fungus-killed flies had an overall appearance slightly different from that of the flies killed by exposure to cold (Ctrl or LC). Their abdomen was more stunted and parched than the cold-killed ones (Figure S1). In addition, by weighing 20 flies, we found that fungus-killed flies were two times lighter than cold-killed ones (Wilcoxon test: W = 356, *p* < 0.001, Figure S2). 

One hour before being used for the experiment, dead flies were individually marked by covering their eyes with a dot of enamel paint (Edding™) (Figure S1). Such an individual marking allowed us to track the location of each fly inside the nest over the course of the experiment.

### 2.5. Experimental Design

Out of the seven queenright colonies collected in the field, we made nine experimental queenless colonies of *Myrmica rubra* (Table S1) containing around 200 workers and 50 larvae (second and third-instar). The population of these nine colonies were standardized by picking up 120 workers inside the test-tube nests and 80 workers walking on the foraging area. Each experimental colony was placed in a tray (22 × 14 × 9 cm) covered with a fine layer of plaster and was allowed to settle in a circular nest (8 cm diameter) that was placed at 5 cm from the borders of the foraging area. The walls and the ceiling of this circular nest were made of laser-cut Plexiglas. The 2 mm height of the nest prevented the overlapping of ant bodies. The resulting monolayer of workers facilitated the observation of their behaviour inside the nest. The nest was periodically moistened by capillarity through a hole created in the plaster layer beforehand, ensuring a satisfactory relative humidity inside, and favouring the aggregation of workers into a large cluster. 

In order to enhance foraging activity, we starved colonies before the experiment by depriving them of food for three days. The experiment started with the introduction of 10 marked flies in the foraging area of the starved ant colonies. As soon as these prey were placed at 15 cm from the nest entrance, we video recorded their retrieval from the food source (with a Logitech^TM^ Webcam C920, Lausanne, Switzerland) as well as the ant behaviour inside the nest (with a Panasonic^TM^ Lumix GH5 camera, Kadoma, Japan) during three hours. After three hours of foraging, we removed the flies that were left over the area, and following the method of Lacey [56], we put them in a thermostatic cabinet in order to check for the emergence of sporulating structures in the following days. After the 3-h foraging, two cameras took a snapshot of the nest interior and the foraging area, every 15 min during 21 h, in order to check for the consumption of the retrieved prey. Then, we reintroduced a sugar solution (0.3 M) feeder and mealworms, on which the colony could feed for 24 h. After a new starvation period of three days, the previously tested colony was offered 10 conidia-free cold-killed prey (Ctrl) and its retrieval response was quantified as described above. This second session of foraging allowed us to test whether the retrieval of conidia-free prey was influenced by a previous exposure of the ant colony to infectious prey. All queenless colonies were tested in each of the three experimental conditions in a random order (Table S1). Between each experimental condition, we waited at least 10 days before assigning the colony to the following condition and during this period we provided food ad libitum to colonies.

For each colony, we counted the number of ants located inside the nest (vs. those on the foraging area) before the start of the experiment as well as 3 h later, at the end of the foraging session. By doing so, we wanted to check that differences in the management of food items were not due to differences in the workforce available inside the nest and whether exposure to infectious prey had led (or not) to some relocation of the nest population at the end of experiment.

### 2.6. Data Collected on Video Recordings

On video recordings, we noted the time at which prey were retrieved inside the nest as well as their possible removal. This allowed to quantify the length of time each fly remained inside the nest.

In each experiment, we followed six flies from their retrieval inside the nest until 90 min later, and we noted every 10 min the number of ants contacting them. In total, 51 Ctrl, 51 LC and 34 Fkill flies were followed inside the nest.

We also counted the number of extensive groomings (EG) displayed by internal workers during three 5-min observations. An extensive grooming included not only the usual grooming of ant antennae and legs but also a bending of the ant gaster followed by an anal-mouth contact (Figure S3). The first observation (basal) was made at the beginning of the experiment when prey were not retrieved yet. The second and third observations were made when the first prey entered the nest (first) and twenty minutes later (twenty), respectively. The number of observed extensive groomings was then weighted by the total number of ants inside the nest observed at the beginning of the experiment. 

Finally, across six colonies, we followed the location of 26 Ctrl, 21 LC, 16 Fkill flies inside the nest, from their entrance until 30 min later. By dividing the nest into 40 quadrants (Figure S4), we identified areas in which larvae were located and the observed flies were deposited by workers. Then, we counted the number of prey relocation from one quadrant to another as well as the length of time dead flies remained in the same quadrants as larvae. 

### 2.7. Statistical Analyses

All data were analysed with R software (version 3.6.2) and all tests were two-tailed with a significance level of alpha = 0.05. As the number of ants inside the nest was normally distributed (Shapiro test: SW = 0.96, *p* = 0.075), we performed a linear mixed model to test the effect of the experimental conditions (i.e., Ctrl prey, LC prey and Fkill prey) and of food introduction (i.e., before and after 3 h of foraging). We included the colonial identity (ID) as a random factor (Table S2). 

The retrieval dynamics of flies inside the nest were compared with Cox proportional hazards regression model by using the R-packages “*survival*” [57] and “*Coxme*” [58]. The prey infectiousness was included as a fixed factor while the colonial ID was included as a random factor. Flies that were not retrieved during the allocated time were included in the database by being assigned the maximal duration of 3 h. The number of flies retrieved inside the nest *(Poisson* distribution, Table S2), the percentage of discarded prey (*binomial* distribution) and the length of time dead flies remained inside the nest (*Gamma distribution*) were analysed using general linear mixed models (GLMM). For the flies that were partially or totally consumed, we considered the time elapsed from their entrance into the nest until the end of the foraging session. For each foraging session, the global level of nest exposure to hazardous prey items was assessed by summing the length of time that all these flies remained inside the nest. For those GLMMs, we included the status of fly prey as a fixed factor and the colonial ID as a random factor. We also performed a GLMM to test the effects of prey status and time on the number of ants contacting flies inside the nest (Poisson distribution). Finally, we used a GLMM (negative binomial distribution) to test the effect of prey type on the number of between-quadrant relocations. 

We carried out LMM and GLMMs with the R-package “*lme4*” [59] to analyse data that met the models’ assumption and that showed no under or over-dispersion based on model deviance/degrees of freedom values. Prior to analysis, the length of time that flies remained inside the nest was transformed with a 1/X transformation to meet the assumptions of the GLMM. To select the best fitted model based on the AIC, we used the R-package “*MuMIn*” [60] with the “*dredge”* function. The significance of fixed factors was tested using the Wald-test with the R-package “*car*” [61]. When significant, we conducted post hoc comparisons by using Tukey tests and Bonferroni adjustment with the R-package “*emmeans*” [62].

In terms of non-parametric tests, we used Friedman tests to check whether (1) the global level of nest exposure to infectious prey, (2) the probability of prey retrieval differed depending on the infectious state of the offered prey. For each colony, we compared the weighted number of extensive grooming (EG) between the observation sessions (basal vs. first vs. twenty) with Friedman tests. To compare the weighted number of EG across the different prey types, we performed Kruskal–Wallis tests for each observation session. Finally, the length of time that flies remained in the same quadrants as larvae was compared across the tested conditions by using a Kruskal–Wallis test. 

The Friedman and Kruskal–Wallis tests were performed using the R-package “*agricolae*” and when significant, we used Fisher’s least significant difference (LSD) test for pairwise post hoc comparisons implemented in the same package.

All data were expressed as medians and the 1st and 3rd quartiles (median [Q1, Q3]), except for the number of ants contacting the retrieved flies as well as the length of time these flies were in the same quadrant as larvae, which were averaged. All the figures were achieved with the package “*ggplot2*” [63].

## 3. Results

Around 110 workers [100,140] were located inside the nest at the beginning of the experiment. The nest population did not differ between the tested conditions (Effect of prey infectiousness: LMM: F test: F = 1.75, *df* = 2, *p =* 0.19) and was not influenced by the introduction of prey (before vs. after three hours of foraging: LMM: F test: F = 0.24, *df* = 1, *p =* 0.63). The interaction effect between these two factors was also not significant (LMM: F test: F = 0.111, *df* = 2, *p =* 0.89).

**Figure 1:**
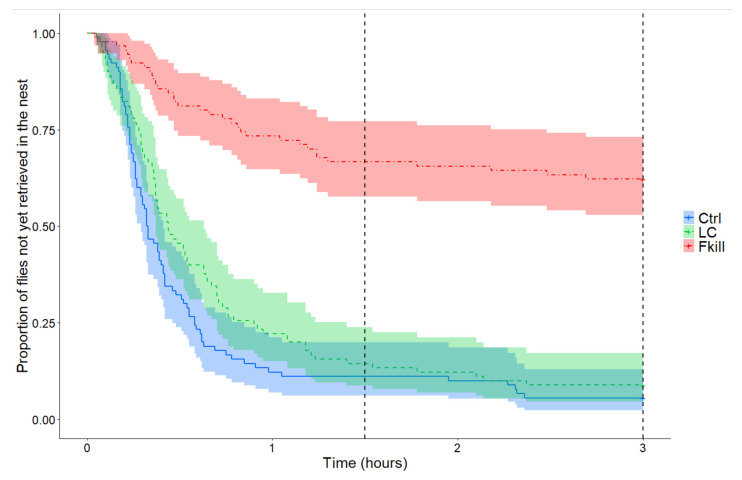
Proportion of flies not yet retrieved inside the nest as a function of time elapsed since prey introduction on the foraging area. The two vertical dashed lines indicate the half (i.e., 1 h 30) and the end of the experiment (i.e., 3 h). Curves are drawn for the different experimental conditions. Blue solid curve: control conidia-free flies (Ctrl, n = 90); green dotted curve: conidia-covered flies (LC, n = 90); red dashed curve: flies killed by the entomopathogenic fungus (Fkill, n = 90); The coloured shade around curves represents the 95% confidence intervals for each condition.

The dynamics of prey retrieval inside the nest differed depending on the offered flies (Cox mixed-effects model: Wald test: χ^2^ = 108.6, *df* = 2, *p* < 0.0001; Figure 1). Control flies were retrieved faster than prey that were covered with conidia (HR = 0.70, *p* = 0.04) or that recently died from fungal infection (HR = 0.09, *p* < 0.0001). Thus, after three hours of foraging, the number of flies that were retrieved by ants differed significantly between conditions (GLMM: Wald test: W = 29.9, *df* = 2, *p* < 0.0001). All the control or conidia-covered prey had been collected in, respectively, eight and seven out of the nine tested colonies (Tukey post hoc test: m_Ctrl_ = m_LC_ = 10 flies [10,10], *p* = 1, Table 1). By contrast, ants were far less eager to take a prey that had recently died from fungal infection. A significantly lower number of fungus-killed flies were thus retrieved inside the nest than for the two other conditions (Tukey post hoc test: m_Fkill_ = 4 flies [1,6], Ctrl vs. Fkill: *p* < 0.0001; LC vs. Fkill: *p* < 0.0001). As a correlate, the probability of retrieving a fly per unit of time differed significantly between conditions (Friedman test: F = 11.63 *df* = 2, *p* = 0.0048). Fungus-killed flies were less likely to be retrieved than control or conidia-covered flies (LSD tests: m_Ctrl_ = 0.046 fly/min [0.043,0.074] vs. m_Fkill_ = 0.003 fly/min [0.0005,0.0051], *p* = 0.0007; m_LC_ = 0.052 fly/min [0.017,0.077] vs. m_Fkill_ = 0.003 fly/min [0.0005,0.0051], *p* = 0.0007, N_Ctrl_ = N_LC_ = N_Fkill_ = 9). 

Foragers thus achieved a first sanitary control by deciding not to retrieve half of the fungus-killed flies. For the fungus-killed prey that were nevertheless retrieved, we investigated how ant workers coped with this potential sanitary risk inside the nest. During the allocated time (i.e., 3 h), we found that the percentage of retrieved flies that were not consumed and that were rejected outside the nest depended on the tested condition (GLMM: Wald test: W = 22.5, *df* = 2, *p* < 0.0001, Table 1). In total, a small percentage of the retrieved control flies (7%, n = 85) or of the conidia-covered prey (11%, n = 82) were discarded outside of the nest (Tukey post-hoc test: Ctrl vs. LC condition, *p* = 1). In contrast, in the case of fungus-killed flies, around half (42%; n = 34) of the retrieved prey were ultimately rejected outside of the nest, this percentage being significantly higher than for the other two experimental conditions (Tukey post hoc test: Fkill vs. LC, *p* = 0.0002; Fkill vs. Ctrl, *p* = 0.0001). In some cases, the rejected flies were brought back inside the nest by a forager. Such a back and forth movement was observed mainly for the Ctrl flies (two out to six rejected flies) and for LC flies (three out of five rejected flies) but exceptionally for the Fkill flies (one out of the fifteen rejected flies). 

**Figure 2:**
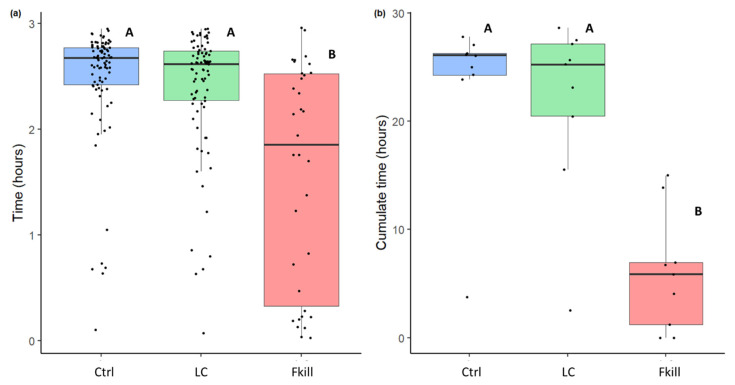
(**a**) Length of time that dead flies remained inside the nest depending on their level of infection. Blue: control flies (n = 85), green: flies covered with conidia (n = 82), red: fungus-killed flies (n = 34). (**b**) Global exposure of ant colonies to prey items. This exposure level is the cumulated lengths of time that all flies remained inside the nest. Blue: control flies (n = 9), green: flies covered with conidia (n = 9), red: fungus-killed flies (n = 9). The horizontal bar within the boxes represents the median; the upper and lower boundaries of the boxes represent, respectively, the 75th and 25th percentiles, while the whiskers extend to the smallest and largest values within 1.5 box lengths. Groups sharing a common letter were not significantly different.

The length of time that flies remained inside the nest, and the number of ants in contact with them allow us to assess the level of food acceptance and the investment of ants in prey handling and management. The flies remained inside the nest for different durations depending on their infectiousness (GLMM: Wald test: W = 127, *df* = 2, *p* < 0.0001). Fungus-killed flies spent less time inside the nest than the control prey (mFkill = 111 min [20,151] vs. mCtrl = 160 min [145,166], Tukey post hoc test: *p* < 0.0001, N_Fkill_ = 34, N_Ctrl_ = 85, Figure 2a) or than the prey covered with conidia (m_Fkill_ = 111 min [20,151] vs. mLC= 157 min [137,164], Tukey post hoc test: *p* < 0.0001, NLC = 82). Regardless of the presence of conidia over their cuticle, cold-killed flies were left for a similar duration inside the nest (mCtrl = 160 min [145,166] vs. mLC = 157 min [137,164], Tukey post hoc test: *p* = 1). The global level of nestmate exposure to prey items also differed depending on their infectious potential (Friedman test: F = 8.67 *df* = 2, *p* =0.013, Figure 2b). Globally, ant colonies were less exposed to fungus-killed prey than to control and conidia-covered prey (LSD test: Fkill vs. LC: *p* = 0.002; Fkill vs. Ctrl: *p* = 0.01, N_Fkill_ = N_Ctrl_ = N_LC_ = 9). This level of exposure was not influenced by the presence of a small amount of conidia on cold-killed flies (LSD test: LC vs. Ctrl: *p* = 0.36). 

**Figure 3:**
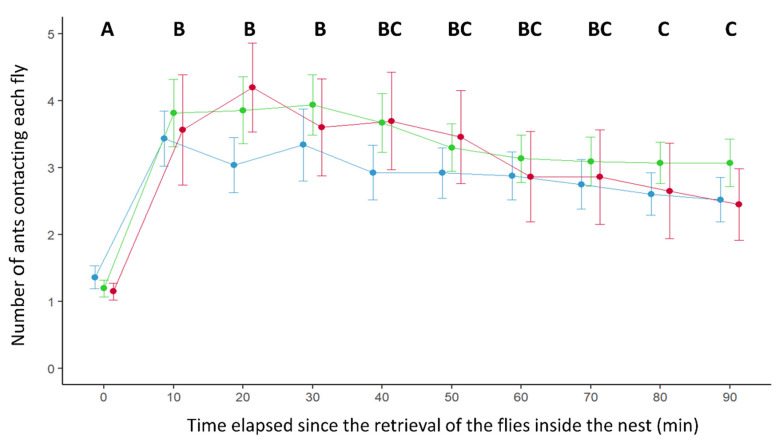
Number of ants contacting each fly inside the nest as a function of the time elapsed since its retrieval. Blue: control flies (n = 51), green: conidia-covered flies (n = 51), red: flies killed by the entomopathogenic fungus (n = 34). The points represent the mean; the bars represent confidence intervals (95%) for each condition. Time steps sharing a common letter were not significantly different.

In terms of interest for the retrieved items, the number of ants surrounding each prey changed with the time elapsed since its introduction inside the nest (Time effect: GLMM: Wald test, W = 187, *df* = 9, *p* < 0.0001). For all the tested conditions, the number of ants in contact with a fly first increased between 10 and 30 min after its retrieval inside the nest, and then progressively declined over time (Figure 3). Noticeably, the number of ants contacting a prey was influenced by its infectious potential (effect of prey infectiousness: GLMM: Wald test: W = 13.05, *df* = 2, *p* = 0.0015). A lower number of ants surrounded the conidia-free flies than the contaminated ones (Figure 3). This difference was, however, significant only for the comparison between conidia-covered flies and control flies (Tukey post hoc test: Ctrl vs. LC, *p* = 0.0013; Ctrl vs. Fkill, *p* = 0.099; LC vs. Fkill *p* = 1). 

Each retrieved fly was deposited and relocated in a number of quadrants that differed depending on its level of infection (GLMM: Wald test: W = 6.32, *df* = 2, *p* = 0.042). Indeed, conidia-free flies were relocated in a lower number of quadrants than fungus-killed flies and conidia-covered flies (m_Ctrl_ = 4.5 quadrants [3,6], NCtrl = 26, mFkill = 6 quadrants [4,10], NFkill = 16, mLC = 7 quadrants [4,8], NLC = 21). Moreover, we found that fungus-killed prey remained in the same quadrant as larvae for the shortest duration compared to the control and conidia-covered flies, even though this difference was not significant (m_Ctrl_ = 206 ± 178 s vs. m_LC_ = 214 ± 149 s vs. m_Fkill_ = 83 ± 144 s; Kruskal–Wallis: KW = 3.85, *df* = 2, *p* = 0.15). 

**Figure 4:**
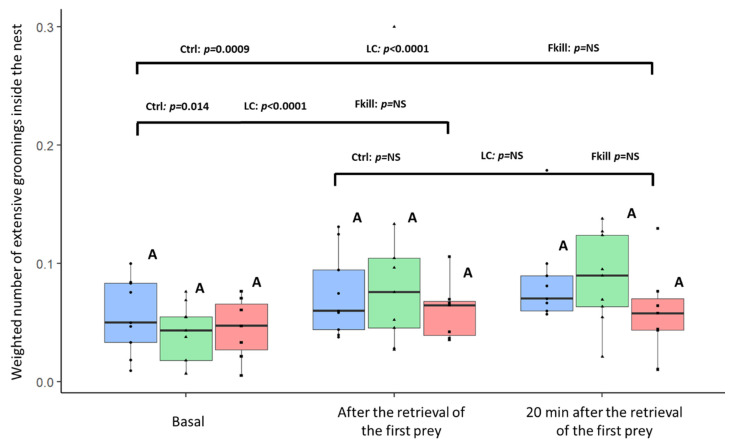
Weighted number of extensive groomings (EG) inside the nest before the introduction of food (basal), after the retrieval of the first prey inside the nest and twenty minutes later. The weighted number was calculated by summing the number of EG within 5 min divided by the number of ants inside the nest. Blue: control flies (n = 27), green: conidia-covered flies (n = 27), red: fungus-killed flies (n = 21). The horizontal bar within the boxes represents the median; the upper and lower boundaries of the boxes represent respectively the 75th and 25th percentiles, while the whiskers extend to the smallest and largest values within 1.5 box lengths. For each condition, *p*-values are given for pairwise comparisons between observation sessions (NS = non-significant, *p* > 0.05). Within each observation session, conditions sharing a common letter were not significantly different.

During three sessions of 5-min observations, we counted the number of extensive groomings (EG) performed by workers inside the nest and close to its entrance. In total, we observed 600 extensive groomings across all the experiments. When foraging on control flies, the weighted number of EG increased as soon as the first prey entered the nest (first) as well as twenty minutes later (Ctrl condition: Friedman: F = 9.31, *df* = 2, *p* = 0.003, post-hoc LSD: basal vs. first: *p* = 0.014; basal vs. twenty: *p* = 0.0009; first vs. twenty: *p* = 0.21; Figure 4). Likewise, there was an increase in the number of EG following the first retrieval of conidia-covered prey (LC condition: Friedman: F = 13.56, *df* = 2, *p* < 0.0001, post hoc LSD: basal vs. first: *p* < 0.0001; basal vs. twenty: *p* < 0.0001; first vs. twenty: *p* = 0.67, N_basal_ = N_first_ = N_twenty_ = 25). However, in the case of fungus-killed prey, the very low number of flies retrieved inside the nest did not induce a significant increase in the EG. (Friedman: F = 2.89, *df* = 2, *p* = 0.25). For each observation session, we did not find any difference in EG depending on the prey infectiousness (Kruskal–Wallis; *df* = 2; Basal: KW = 1.19, *p* = 0.55; First: KW = 0.75 *p* = 0.69; Twenty: KW = 2.7, *p* = 0.26). The infectious potential of the retrieved items did not seem to influence the performance of extensive groomings by internal workers.

Finally, we tested whether ant colonies having previously foraged on fungus-contaminated prey changed their willingness to further retrieve the same—but conidia-free—prey item. On a second foraging round, the number of conidia-free flies that were retrieved by foragers did not differ according to their previous foraging experience (Friedman test: F = 0, *df* = 2, *p* = 1). Across all colonies, out of the 90 offered flies, 97.8%, 100% and 93.4% were retrieved when colonies had previously foraged on control flies, conidia-covered flies and fungus-killed flies, respectively. 

## 4. Discussion

When ants forage for food resources, the detection and discrimination of infected items is crucial to avoid the uptake of pathogens [10,37], especially when the food item is not consumed on the spot but physically retrieved inside the nest or even stored for a while before being eaten. This is namely the case for leaf-cutting and harvester ants, which respectively retrieve leaves for the growth of a mutualistic fungus [64,65] and collect seeds to be stored in granaries or consumed by nestmates [66]. The retrieval of whole food items is also achieved by hunting and scavenging ants [67], for which the insect cadavers brought into the nest may become hazardous for the colony survival, especially if prey were diseased or killed by entomopathogens [4]. As regards the behaviour of *M. rubra* foragers, they slightly discriminate prey even when the associated pathogenicity is low, as is the case for prey bearing a small amount of conidia. A more clear-cut discrimination occurred for prey with a delayed pathogenicity, as is the case for recently fungus-killed prey.

In the first case of prey covered with a low amount of entomopathogenic *M. brunneum* conidia, the dynamics of retrieval was slower than when the prey were not contaminated. Thus, foragers were less hasty to retrieve a slightly infectious prey than a non-pathogenic one. However, once being retrieved by foragers inside the nest, almost none of the conidia-covered flies were discarded and most prey were eventually consumed within 24 h. Discrimination of prey with a low conidia load mainly took place in the outside and was achieved by the ant foragers. This finding confirms that foragers, which are the first ones to discover dead insects, act as a first line of defence against pathogens by hesitating to retrieve potentially hazardous food items. The protective behaviour of foragers thus ranges from a clear-cut avoidance of heavily sporulating prey [35,38] to a mere slowdown of retrieval of slightly infectious prey, as shown in the current study. From a functional perspective, the slower retrieval of conidia-covered flies allows foragers to explore the discovered food item with their antennae and to assess more accurately the presence of conidia over the insect body [68,69]. Furthermore, foragers were slower to transport a conidia-covered prey because they spent more time manipulating and licking this prey on the foraging area. Further studies should assess to what extent this behaviour contributes to the removal of conidia from the prey before its retrieval into the nest.

Another main finding is the ability of ant foragers to discriminate a prey that may become a sanitary threat in the future—i.e., a prey which recently died from fungal infection but not yet sporulating. In this latter case, ant colonies showed a clear-cut avoidance, with most fungus-killed prey remaining in the foraging area and being not retrieved by foragers inside the nest. This observation confirms a previous study on individual foragers which were reluctant to pick up fungus-killed prey [38]. More broadly, this is in line with other studies in termites showing that workers do not cannibalize fungus-killed nestmates [36]. By refraining from taking such prey, even before the emergence of conidiophores, foragers implement a prophylactic strategy that prevents the exposure of nestmates to a future source of infection. Such a prophylactic strategy was not generalized during further foraging bouts on the same type of prey. Indeed, we found that ant colonies that had been offered fungus-killed flies were as eager to retrieve such prey, for a second time, when they were no longer contaminated (see also [38]). In nature, ant colonies feed on dead insects several times over successive foraging days [70]. In the case of generalist scavengers such as *M. rubra* ants, a generalization of disease-related cues to all prey, including “safe” ones, would lead to a lower number of retrieved cadavers and to a useless reduction in their diet range.

To date, we do not know whether the low retrieval of fungus-killed insects reflects a loss of interest for a low-quality food item or a pathogen-specific sanitary strategy. A disinterest for fungus-killed insects may come from the small amount of body fluids and tissue that are still available for consumption by the ants. Indeed, just before the death of the infected fly, the fungal hyphae had grown and colonized its haemocoel, thereby probably modifying its palatability for ant scavengers [71,72]. Nevertheless, a few fungus-killed prey were retrieved in the nest. Unlike inert items which are straight discarded out of the nest (personal observations), fungus-killed flies raised the interest of internal workers that contacted them for prolonged duration, suggesting that they might still be considered as a food source. The low retrieval of fungus-killed prey could also reflect changes in the composition of the insect haemolymph. Indeed, diglycerides such as 1,2-diolein, which are the major lipid component of insect haemolymph [73], are known to attract and promote removal behaviour in ants [74,75]. Likewise, free fatty acids such as oleic acid make items be treated as food [76] or induce transport behaviour in ants [77]. Further studies should quantify to what extent fungal hyphae in the haemocoel alter the nutritive content but also the composition of insect haemolymph, in particular its fatty acid components. A last explanation is that the avoidance of fungus-killed prey by foragers results from their perception of disease-induced changes in the prey chemical profile [40,78,79]. For instance, cuticular hydrocarbons are good candidates as cues for the pathogenicity of prey since they can be perceived by ants and are relevant in interactions between species, in contrast to other, non-hydrocarbon substances [80,81]. As already found in several social insects which can detect sick nestmates from healthy ones (ants: [11,48], bees: [82], termites: [52,83]), one may assume that workers can perceive the presence of entomopathogens having caused the death of a potential prey. Further investigations are needed to identify the specific compounds emitted by *M. bruneum* fungus—and by other types of pathogens—that are detected by the olfactory and/or gustatory ant sensillae and that trigger food avoidance among foragers. 

Once being retrieved into the nest, the prey were handled in different ways depending on their associated sanitary risk. For the conidia-free prey, the fly’s wings were discarded within minutes, while the thorax and abdomen were butchered into smaller pieces. Since large food items cannot transit towards the mid-gut, where they could be digested, butchering appears as a compulsory step that allows ants to ingest body fluids and haemolymph of prey. Prey fragments can also be put on larvae which directly feed upon or externally digest prey tissue for consumption by other colony members [1,84]. Furthermore, since the larvae of social insects, in particular ants and wasps, have different digestive enzymes than adults, they may potentially help in sanitizing contaminated food [84,85,86]. However, in our study, conidia-free flies were put in the same area as larvae only for a short duration, suggesting that the butchering process and digestion of prey was mainly achieved by *M. rubra* ant workers instead of larvae [1,84].

As for the conidia-covered prey, they were located for a similar duration near brood and they elicited more inspection by internal workers than conidia-free flies. Such an enhanced manipulation of conidia-covered prey could contribute to the removal of conidia from their cuticle before being fed upon by the ants. Concerning the few retrieved fungus-killed flies, they elicit quite different responses among ant workers which deposited them close to larvae for twice less time compared to conidia-free prey. Internal workers seem to manage fungus-killed flies as potentially harmful, or at least poorly nutritive food items. Furthermore, fungus-killed flies underwent the most frequent relocations inside the nest before being ultimately discarded.

Noticeably, in some occasions, we observed back-and forth transports of fungus-killed prey that were alternatively discarded and retrieved again inside the nest. This suggests that internal workers may “disagree” about the sanitary status and/or nutritive value of the fungus-killed prey. This could be due to interindividual differences in the detection threshold of fungus-induced changes on prey. This suggests that foragers may differ from other inner workers in their response threshold to prey chemical cues, as already reported for colonial odour or the recruitment trail [87,88].

Besides the foragers that ensure a first line of defence against the uptake of pathogens, we found that nestmates sort out prey items according to their sanitary status by discarding fungus-killed prey outside of the nest. For the conidia-covered prey, the infrabuccal pocket may act as a filter that prevent conidia from spreading in the digestive tract of the ants [89,90]. Furthermore, ant workers performed intensive self-groomings interspersed by an anal-mouth contact through the bending of their gaster. A similar behaviour has been reported as a so-called acidopore grooming in several formicinae species (*Lasius neglectus*: [91]; *Camponotus floridanus*: [92]) or as a faecal fluid grooming in *Atta* ants [93]. In formicinae ants, acidopore grooming was related to the release of poison secretions, which limit the growth of pathogens on the ant’s cuticle [91]. Ants swallow acidic poison gland secretions which act as microbial control agents in the gut and that sanitize ingested food [92]. In *Atta spp* ants, faecal droplets collected while grooming were used by the foundress to facilitate the removal or decontamination of particles remaining on the ant’s body or in the fungus garden [93]. In *Myrmica* ants, which lacks acidopore and does not produce formic acid, extensive grooming with anal-mouth contacts might reinforce protection against the development of pathogens in the gut or over the cuticle. We found that this behaviour increased concurrently with the colonial activity that was enhanced by the introduction of food items inside the nest. Since extensive self-grooming by *M. rubra* ants did not differ with the infectiousness of the retrieved prey, it rather appears as a non-specific prophylactic behaviour similar to the “washing hands before dinner” hygienic measure. Our observations are in line with Tragust et al. 2020 study [92] who found a higher proportion of acidopore grooming after fluid ingestion, irrespective of the fluid nutritional value.

## 5. Conclusions

In conclusion, food safety and colony protection against pathogens are ensured by sanitary filters that vary depending on the developmental stage of *Metarhizium brunneum* fungus. First, foragers avoid the uptake of pathogen-killed prey and/or delay the retrieval of conidia-bearing items. Second, hazardous items that nevertheless enter the nest are carefully inspected and may be ultimately rejected by internal workers acting as second-line health auditors. The possible role of larvae in waste management (Pereira et al., 2020, in press) and in the early detection of sanitary risks still remains to be investigated. Unlike predatory or aphid-tending ants, scavenger ants forage on insect cadavers, which may have died due to pathogens, whose diversity is potentially high. In order to discriminate a “safe” prey from a “potentially harmful” prey, it seems unlikely that generalist scavengers such as ants can recognize the specific odours induced by all possible insect parasites. A more plausible explanation is that ant scavengers disregard pathogen-killed insects due to a loss of nutritive content and/or ‘general prey cue’ such as the fatty acids shared by most living insects. Surprisingly, little is known on how generalist scavengers or predators use allelochemical cues to assess prey characteristics [94,95,96] but this question opens up interesting perspectives in the context of pathogen avoidance in insect societies.

## Figures and Tables

**Table 1:** Prey retrieval and prey discarding as a function of their infectious state. For the retrieved flies, median, first and third quartiles values are given. The discarded flies are expressed in percentage. After general linear mixed models (GLMMs), Tukey post hoc tests were made with Bonferroni correction. Values sharing a common letter were not significantly different.

	Control Prey	Conidia-Covered Prey	Fungus-Killed Prey
**Number of Flies Retrieved Inside the Nest**	10 [10,10] ^A^ (n = 9)	10 [10,10] ^A^ (n = 9)	4 [1,6] ^B^ (n = 9)
	GLMM: Wald test: W = 29.9, *df* = 2, *p* < 0.0001		
**Percentage of Discarded Flies**	7% ^A^ (n = 85)	11% ^A^ (n = 82)	42% ^B^ (n = 34)
	GLMM: Wald test: W = 22.5, *df* = 2, *p* < 0.0001		

## Data Availability

Data used in this article are available in the Zenodo Digital Repository: http://doi.org/10.5281/zenodo.3942154.

## Supplementary Materials

The following are available online at https://www.mdpi.com/2075-4450/11/7/444/s1. Figure S1: (A) flies killed by exposure to cold and (B) flies killed by the entomopathogenic fungus less than 24 hours ago. Figure S2: Weight (mg) of prey killed by exposure to cold (N=20, blue) and killed by Metarhizium brunneum (N=20, red). Figure S3: Picture of an ant performing an “extensive grooming”. Figure S4: Picture of the nest taken from above. Table S1: Information about the identity of queenright colonies collected in the field, the geolocalisation of their collection, and the identity of queenless colonies created for the experiment. Table S2: Description of statistical models used in the manuscript.

## References

[1] Hölldobler B., Wilson E.O. (1990). The Ants.

[2] Duarte A., Weissing F.J., Pen I., Keller L. (2011). An evolutionary perspective on self-organized division of labor in social insects. Annu. Rev. Ecol. Evol. Syst..

[3] Schmid-Hempel P. (1998). Parasites in Social Insects.

[4] Boomsma J.J., Schmid-Hempel P., Hughes W.O.H. (2005). Life histories and parasite pressure across the major groups of social insects. Insect Evolutionary Ecology.

[5] Siva-Jothy M.T., Moret Y., Rolff J. (2005). Insect immunity: An evolutionary ecology perspective. Advances in Insect Physiology.

[6] Tsakas S., Marmaras V.J. (2010). Insect immunity and its signalling: An overview. Invertebr. Surviv. J..

[7] Cremer S., Armitage S.A.O., Schmid-Hempel P. (2007). Social immunity. Curr. Biol..

[8] Cremer S. (2019). Social immunity in insects. Curr. Biol..

[9] Meunier J. (2015). Social immunity and the evolution of group living in insects. Philos. Trans. R. Soc. B Biol. Sci..

[10] Cremer S., Pull C.D., Fürst M.A. (2017). Social immunity: Emergence and evolution of colony-level disease protection. Annu. Rev. Entomol..

[11] Konrad M., Pull C.D., Metzler S., Seif K., Naderlinger E., Grasse A.V., Cremer S. (2018). Ants avoid superinfections by performing risk-adjusted sanitary care. Proc. Natl. Acad. Sci. USA.

[12] Calleri D.V., Rosengaus R.B., Traniello J.F.A. (2010). Disease resistance in the drywood termite, *Incisitermes schwarzi*: Does nesting ecology affect immunocompetence?. J. Insect Sci..

[13] Traniello J.F., Rosengaus R.B., Savoie K. (2002). The development of immunity in a social insect: Evidence for the group facilitation of disease resistance. Proc. Natl. Acad. Sci. USA.

[14] Evans J.D., Spivak M. (2010). Socialized medicine: Individual and communal disease barriers in honey bees. J. Invertebr. Pathol..

[15] Hoggard S.J., Wilson P.D., Beattie A.J., Stow A.J. (2011). Social complexity and nesting habits are factors in the evolution of antimicrobial defences in wasps. PLoS ONE.

[16] Liu L., Zhao X.-Y., Tang Q.-B., Lei C.-L., Huang Q.-Y. (2019). The mechanisms of social immunity against fungal infections in eusocial insects. Toxins.

[17] Wilson-Rich N., Spivak M., Fefferman N.H., Starks P.T. (2009). Genetic, individual, and group facilitation of disease resistance in insect societies. Annu. Rev. Entomol..

[18] Stroeymeyt N., Grasse A.V., Crespi A., Mersch D.P., Cremer S., Keller L. (2018). Social network plasticity decreases disease transmission in a eusocial insect. Science.

[19] Naug D., Smith B. (2007). Experimentally induced change in infectious period affects transmission dynamics in a social group. Proc. R. Soc. B Biol. Sci..

[20] Stroeymeyt N., Casillas-Pérez B., Cremer S. (2014). Organisational immunity in social insects. Curr. Opin. Insect Sci..

[21] Verza S.S., Diniz E.A., Chiarelli M.F., Mussury R.M., Bueno O.C. (2017). Waste of leaf-cutting ants: Disposal, nest structure, and abiotic soil factors around internal waste chambers. Acta Ethol..

[22] Weiss M.R. (2006). Defecation Behavior and Ecology of Insects. Annu. Rev. Entomol..

[23] Diez L., Deneubourg J.-L., Detrain C. (2012). Social prophylaxis through distant corpse removal in ants. Naturwissenschaften.

[24] Diez L., Lejeune P., Detrain C. (2014). Keep the nest clean: Survival advantages of corpse removal in ants. Biol. Lett..

[25] Diez L., Urbain L., Lejeune P., Detrain C. (2015). Emergency measures: Adaptive response to pathogen intrusion in the ant nest. Behav. Process..

[26] Visscher P.K. (1983). The honey bee way of death: Necrophoric behaviour in *Apis mellifera* colonies. Anim. Behav..

[27] Wen P. (2020). Death recognition by undertaker bees. bioRxiv.

[28] Munday Z., Brown M.J.F. (2018). Bring out your dead: Quantifying corpse removal in *Bombus terrestris*, an annual eusocial insect. Anim. Behav..

[29] López-Riquelme G.O., Fanjul-Moles M.L. (2013). The funeral ways of social insects. Social strategies for corpse disposal. Trends Entomol..

[30] Sun Q., Zhou X. (2013). Corpse management in social insects. Int. J. Biol. Sci..

[31] Heinze J., Walter B. (2010). Moribund ants leave their nests to die in social isolation. Curr. Biol..

[32] Leclerc J.-B., Detrain C. (2017). Loss of attraction for social cues leads to fungal-infected *Myrmica rubra* ants withdrawing from the nest. Anim. Behav..

[33] Rueppell O., Hayworth M.K., Ross N.P. (2010). Altruistic self-removal of health-compromised honey bee workers from their hive: Self-removal in honey bees. J. Evol. Biol..

[34] Reber A., Chapuisat M. (2012). Diversity, prevalence and virulence of fungal entomopathogens in colonies of the ant *Formica selysi*. Insectes Sociaux.

[35] Marikovsky P.I. (1962). On some features of behavior of the ants *Formica rufa* L. infected with fungous disease. Insectes Sociaux.

[36] Kramm K.R., West D.F., Rockenbach P.G. (1982). Termite pathogens: Transfer of the entomopathogen *Metarhizium anisopliae* between *Reticulitermes* sp. termites. J. Invertebr. Pathol..

[37] Tranter C., LeFevre L., Evison S.E.F., Hughes W.O.H. (2015). Threat detection: Contextual recognition and response to parasites by ants. Behav. Ecol..

[38] Pereira H., Detrain C. (2020). Pathogen avoidance and prey discrimination in ants. R. Soc. Open Sci..

[39] Hussain A., Tian M.-Y., He Y.-R., Bland J.M., Gu W.-X. (2010). Behavioral and electrophysiological responses of *Coptotermes formosanus Shiraki* towards entomopathogenic fungal volatiles. Biol. Control.

[40] Crespo R., Juarez M.P., Cafferata L.F.R. (2000). Biochemical Interaction between Entomopathogenous Fungi and Their Insect-Host-like Hydrocarbons. Mycologia.

[41] Cheraghi A., Habibpour B., Mossadegh M.S. (2013). Application of Bait Treated with the Entomopathogenic Fungus *Metarhizium anisopliae* (Metsch.) Sorokin for the control of *Microcerotermes diversus* Silv. Psyche J. Entomol..

[42] Pontieri L., Vojvodic S., Graham R., Pedersen J.S., Linksvayer T.A. (2014). Ant colonies prefer infected over uninfected nest sites. PLoS ONE.

[43] Qiu H.-L., Lu L.-H., Zhang C.-Y., He Y.-R. (2014). Pathogenicity of individual isolates of entomopathogenic fungi affects feeding preference of red imported fire ants *Solenopsis invicta*. Biocontrol Sci. Technol..

[44] Leclerc J.-B., Silva J.P., Detrain C. (2018). Impact of soil contamination on the growth and shape of ant nests. R. Soc. Open Sci..

[45] Brian M.V., Abbott A. (1977). The control of food flow in a society of the ant *Myrmica rubra* L.. Anim. Behav..

[46] Ortiz-Urquiza A., Keyhani N.O. (2013). Action on the surface: Entomopathogenic fungi versus the insect cuticle. Insects.

[47] Elmes G.W. (1973). Observations on the density of queens in natural colonies of *Myrmica rubra* L. (*Hymenoptera: Formicidae*). J. Anim. Ecol..

[48] Leclerc J.-B., Detrain C. (2016). Ants detect but do not discriminate diseased workers within their nest. Sci. Nat..

[49] Keller S., Kessler P., Schweizer C. (2003). Distribution of insect pathogenic soil fungi in Switzerland with special reference to *Beauveria brongniartii* and *Metharhizium anisopliae*. Biocontrol.

[50] Goettel M.S., Eilenberg J. (2010). Entomopathogenic fungi and their role in regulation of insect populations. Insect Control: Biological and Synthetic Agents.

[51] Loreto R.G., Hughes D.P. (2016). Disease dynamics in ants. Advances in Genetics.

[52] Davis H.E., Meconcelli S., Radek R., McMahon D.P. (2018). Termites shape their collective behavioural response based on stage of infection. Sci. Rep..

[53] Bulmer M.S., Franco B.A., Fields E.G. (2019). Subterranean termite social alarm and hygienic responses to fungal pathogens. Insects.

[54] Simone-Finstrom M.D., Spivak M. (2012). Increased resin collection after parasite challenge: A case of self-medication in honey bees?. PLoS ONE.

[55] Hänel H. (1982). The life cycle of the insect pathogenic fungus *Metarhizium anisopliae* in the termite *Nasutitermes exitiosus*. Mycopathologia.

[56] Lacey L.A. (2012). Manual of Techniques in Invertebrate Pathology.

[57] Therneau T.M., Grambsch P.M. (2000). Modeling Survival Data: Extending the Cox Model.

[58] Therneau T.M. (2020). Coxme: Mixed Effects Cox Models. https://CRAN.R-project.org/package=coxme.

[59] Bates D., Maechler M., Bolker B., Walker S. (2015). Fitting Linear Mixed-Effects Models Using {lme4}. J. Stat. Softw..

[60] Barton K. (2019). MuMIn: Multi-Model Inference; R package version 1.43.15. https://CRAN.R-project.org/package=MuMIn.

[61] Fox J., Weisberg S. (2019). An R Companion to Applied Regression.

[62] Lenth R. (2020). Emmeans: Estimated Marginal Means, Aka Least-Squares Means. https://CRAN.R-project.org/package=emmeans.

[63] Wickham H. (2016). ggplot2: Elegant Graphics for Data Analysis.

[64] Coblentz K.E., Van Bael S.A. (2013). Field colonies of leaf-cutting ants select plant materials containing low abundances of endophytic fungi. Ecosphere.

[65] Griffiths H.M., Hughes W.O.H. (2010). Hitchhiking and the removal of microbial contaminants by the leaf-cutting ant *Atta colombica*. Ecol. Entomol..

[66] Knoch T.R., Faeth S.H., Arnott D.L. (1993). Endophytic fungi alter foraging and dispersal by desert seed-harvesting ants. Oecologia.

[67] Gayahan G.G., Tschinkel W.R. (2008). Fire ants, *Solenopsis invicta*, dry and store insect pieces for later use. J. Insect Sci..

[68] Yanagawa A., Yokohari F., Shimizu S. (2009). The role of antennae in removing entomopathogenic fungi from cuticle of the termite, *Coptotermes formosanus*. J. Insect Sci..

[69] Meyling N.V., Pell J.K. (2006). Detection and avoidance of an entomopathogenic fungus by a generalist insect predator. Ecol. Entomol..

[70] Carroll C.R., Janzen D.H. (1973). Ecology of Foraging by Ants. Annu. Rev. Ecol. Syst..

[71] Vestergaard S., Butt T.M., Bresciani J., Gillespie A.T., Eilenberg J. (1999). Light and Electron Microscopy Studies of the Infection of the Western Flower *Thrips Frankliniella occidentalis (Thysanoptera: Thripidae)* by the Entomopathogenic Fungus *Metarhizium anisopliae*. J. Invertebr. Pathol..

[72] Butt T.M., Coates C.J., Dubovskiy I.M., Ratcliffe N.A. (2016). Entomopathogenic fungi. Advances in Genetics.

[73] Beenakkers A.M.T., Gilbert L.I. (1968). The fatty acid composition of fat body and haemolymph lipids in Hyalophora cecropia and its relation to lipid release. J. Insect Physiol..

[74] Brew C.R., O’Dowd D.J., Rae I.D. (1989). Seed dispersal by ants: Behaviour-releasing compounds in elaiosomes. Oecologia.

[75] Hughes L., Westoby M., Jurado E. (1994). Convergence of elaiosomes and insect prey: Evidence from ant foraging behaviour and fatty acid composition. Funct. Ecol..

[76] Gordon D.M. (1983). Dependence of necrophoric response to oleic acid on social context in the ant, *Pogonomyrmex badius*. J. Chem. Ecol..

[77] Diez L., Moquet L., Detrain C. (2013). Post-mortem changes in chemical profile and their influence on corpse removal in ants. J. Chem. Ecol..

[78] Morath S.U., Hung R., Bennett J.W. (2012). Fungal volatile organic compounds: A review with emphasis on their biotechnological potential. Fungal Biol. Rev..

[79] Pedrini N. (2018). Molecular interactions between entomopathogenic fungi (Hypocreales) and their insect host: Perspectives from stressful cuticle and hemolymph battlefields and the potential of dual RNA sequencing for future studies. Fungal Biol..

[80] Lang C., Menzel F. (2011). *Lasius niger* ants discriminate aphids based on their cuticular hydrocarbons. Anim. Behav..

[81] Binz H., Foitzik S., Staab F., Menzel F. (2014). The chemistry of competition: Exploitation of heterospecific cues depends on the dominance rank in the community. Anim. Behav..

[82] Baracchi D., Fadda A., Turillazzi S. (2012). Evidence for antiseptic behaviour towards sick adult bees in honey bee colonies. J. Insect Physiol..

[83] Rosengaus R.B., Jordan C., Lefebvre M.L., Traniello J.F.A. (1999). Pathogen alarm behavior in a termite: A new form of communication in social insects. Naturwissenschaften.

[84] Cassill D.L., Butler J., Vinson S.B., Wheeler D.E. (2005). Cooperation during prey digestion between workers and larvae in the ant, *Pheidole spadonia*. Insectes Sociaux.

[85] Sorensen A.A., Kamas R.S., Vinson S.B. (1983). The influence of oral secretions from larvae on levels of proteinases in colony members of *Solenopsis invicta Buren* (Hymenoptera: Formicidae). J. Insect Physiol..

[86] Ishay J., Ikan R. (1968). Food exchange between adults and larvae in *Vespa orientalis* F. Anim. Behav..

[87] Detrain C., Pereira H., Fourcassié V. (2019). Differential responses to chemical cues correlate with task performance in ant foragers. Behav. Ecol. Sociobiol..

[88] Depickère S., Fresneau D., Detrain C., Deneubourg J.-L. (2004). Marking as a decision factor in the choice of a new resting site in *Lasius niger*. Insectes Sociaux.

[89] Glancey B.M., Vander Meer R.K., Glover A., Lofgren C.S., Vinson S.B. (1981). Filtration of microparticles from liquids ingested by the red imported fire ant *Solenopsis invicta Buren*. Insectes Sociaux.

[90] Eisner T., Happ G.M. (1962). The Infrabuccal Pocket of a Formicine ant: A social filtration device. Psyche (Stuttg.).

[91] Tragust S., Mitteregger B., Barone V., Konrad M., Ugelvig L.V., Cremer S. (2013). Ants disinfect fungus-exposed brood by oral uptake and spread of their poison. Curr. Biol..

[92] Tragust S., Herrmann C., Häfner J., Braasch R., Tilgen C., Hoock M., Milidakis M.A., Gross R., Feldhaar H. (2020). Formicine ants swallow their highly acidic poison for gut microbial selection and control. BioRxiv.

[93] Fernández-Marín H., Zimmermann J.K., Wcislo W.T. (2003). Nest-founding in *Acromyrmex octospinosus* (Hymenoptera, Formicidae, Attini): Demography and putative prophylactic behaviors. Insectes Sociaux.

[94] Binz H., Kraft E.F., Entling M.H., Menzel F. (2016). Behavioral response of a generalist predator to chemotactile cues of two taxonomically distinct prey species. Chemoecology.

[95] Buehlmann C., Graham P., Hansson B.S., Knaden M. (2014). Desert ants locate food by combining high sensitivity to food odors with extensive crosswind runs. Curr. Biol..

[96] Pearce-Duvet J.M.C., Feener D.H. (2010). Resource discovery in ant communities: Do food type and quantity matter?. Ecol. Entomol..

