# Supplementary material for: Prophylactic Avoidance of Hazardous Prey by the Ant Host Myrmica rubra
Source: Insects. 2020 Jul 14;11(7):444. doi: 10.3390/insects11070444 (PMC7412340; doi:10.3390/insects11070444)
Supplement: Supplementary file 1 [file insects-11-00444-s001.pdf]

Supplementary material

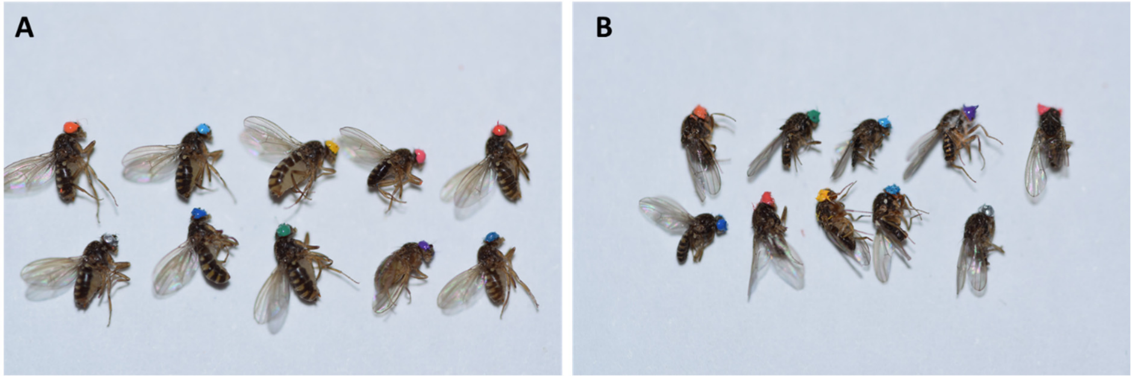

Figure S.1: (A) flies killed by exposure to cold and (B) flies killed by the entomopathogenic fungus less than 24 hours ago. Flies were individually marked by painting a colour dot on their eyes. This picture was taken one hour before their introduction in the foraging area.

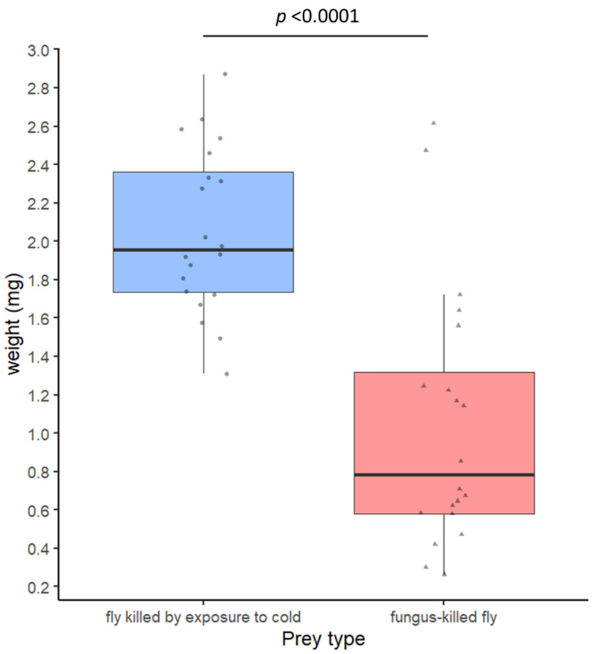

Figure S.2 : Weight (mg) of prey killed by exposure to cold (N=20, blue) and killed by *Metarhizium brunneum* (N=20, red). The horizontal bar within the boxes represents the median; the upper and lower boundaries of the boxes represent respectively the 75th and 25th percentiles, while the whiskers extend to the smallest and largest values within 1.5 box lengths. The *p* value of the Mann-Whitney test is displayed above the two boxplots and indicates that fungus-killed flies are lighter than flies killed by exposure to cold.

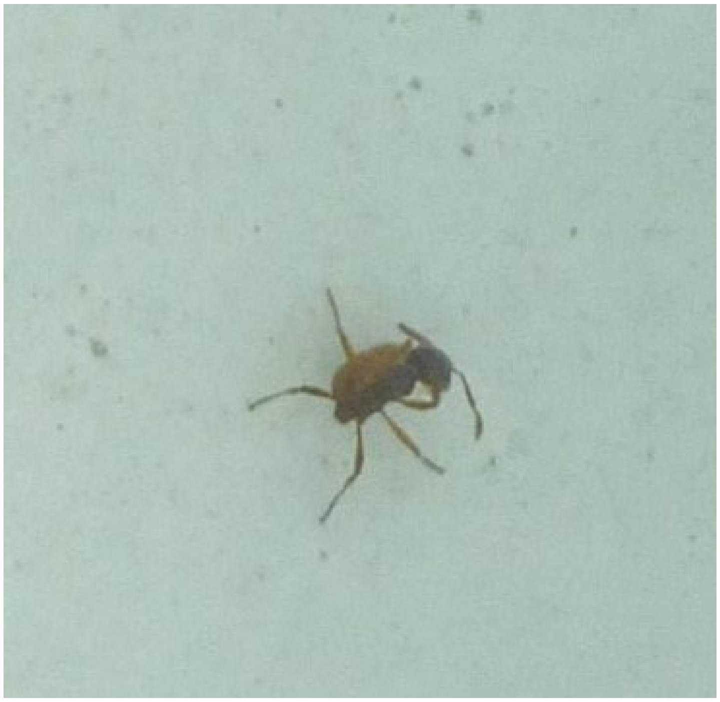

Figure S.3 : Picture of an ant performing an “extensive grooming”. The ant bends its gaster and head to reach its anal zone with its mouth during a grooming event.

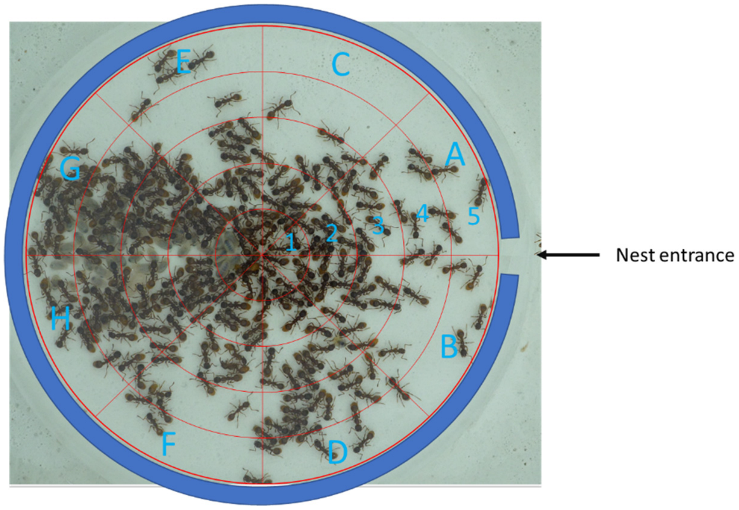

Figure S.4: Picture of the nest taken from above. The 40 quadrants are displayed on the picture. The nest has been divided into 8 large areas (letters), themselves separated into 5 areas (numbers) from the centre to the periphery of the nest.

Table S.1 : Information about the identity of queenright colonies collected in the field, the geolocalisation of their collection, and the identity of queenless colonies created for the experiment. We also give the order of the experimental conditions that each queenless colony had received. Each experimental condition was composed of two different foraging sessions, during the first we introduced either conidia-free flies (Ctrl), conidia-covered (LC) or fungus-killed flies (Fkill). During the second, we always introduced conidia-free Ctrl flies. We waited at least 10 days before assigning the colony to the following experimental condition.

Queenright colonies collected in the field (N=7)	Localities	Geolocalisation (North-East)	Queenless colonies used in the experiment (N=9)	Order of the experimental conditions					
				1st foraging session	2nd foraging session	1st foraging session	2nd foraging session	1st foraging session	2nd foraging session
Col1	FALISOLLE	50.42003, 4.6310092	Col1	LC	Ctrl	Fkill	Ctrl	Ctrl	Ctrl
Col2	AISEAU	50.42756, 4.59365	Col2	LC	Ctrl	Ctrl	Ctrl	Fkill	Ctrl
Col3	AISEAU	50.42818, 4.5953	Col3	LC	Ctrl	Ctrl	Ctrl	Fkill	Ctrl
Col4	AISEAU	50.42696, 4.59443	Col4	Ctrl	Ctrl	Fkill	Ctrl	LC	Ctrl
Col5	BRUSSEL	50.8183791, 4.400238	Col5	Ctrl	Ctrl	Fkill	Ctrl	LC	Ctrl
Col6	FALLISOLE	50.42058, 4.63326	Col6a	Fkill	Ctrl	Ctrl	Ctrl	LC	Ctrl
			Col6b	Ctrl	Ctrl	LC	Ctrl	Fkill	Ctrl
Col7	BRUSSEL	50.8178289, 4.400433	Col7a	Fkill	Ctrl	LC	Ctrl	Ctrl	Ctrl
			Col7b	LC	Ctrl	Ctrl	Ctrl	Fkill	Ctrl

Table S.2: Description of statistical models used in the manuscript

Variable analysed	Fitted distribution	Fixed factors	Random factor
**Number of ants inside the nest**<br>*LMM*	*Gaussian* distribution	prey type<br>*time	Colonial ID
**Number of flies retrieved during the foraging session**<br>*GLMM*	*Poisson* distribution<br>*identity* link	prey type	Colonial ID
**Proportion of flies rejected outside of the nest**<br>*GLMM*	Binomial distribution<br>*probit* link	prey type	Colonial ID
**Time spent by flies inside the nest**<br>*GLMM*	Gamma distribution<br>*inverse* link	prey type	Colonial ID
**Number of ants in contact with focused flies**<br>*GLMM*	*Poisson* distribution<br>*log* link	prey type<br>+time	Colonial ID
**Number of quadrants in which flies were transported**<br>GLMM	Negative binomial	Prey type	Colonial ID
